# Crosstalk between hepatitis B virus X and high‐mobility group box 1 facilitates autophagy in hepatocytes

**DOI:** 10.1002/1878-0261.12165

**Published:** 2018-01-24

**Authors:** Sha Fu, Juan Wang, Xingwang Hu, Rong‐rong Zhou, Yongming Fu, Daolin Tang, Rui Kang, Yan Huang, Lunquan Sun, Ning Li, Xue‐Gong Fan

**Affiliations:** ^1^ Department of Infectious Diseases Hunan Key Laboratory of Viral Hepatitis Xiangya Hospital Central South University Changsha China; ^2^ Department of Surgery University of Pittsburgh PA USA; ^3^ Center for Molecular Medicine Xiangya Hospital Central South University Changsha China; ^4^ Department of Blood Transfusion Xiangya Hospital Central South University Changsha China

**Keywords:** acetylation, autophagy, hepatitis B virus, high‐mobility group box 1, histone deacetylases, protein protein interaction

## Abstract

Hepatitis B virus (HBV) X (HBx) protein is a pivotal regulator of HBV‐triggered autophagy. However, the role of HBx‐induced epigenetic changes in autophagy remains largely unknown. The cytoplasmic (Cyt) high‐mobility group box 1 (HMGB1) has been identified as a positive regulator of autophagy, and its Cyt translocation is closely associated with its acetylation status. Here, we evaluated the function of HMGB1 in HBx‐mediated autophagy and its association with histone deacetylase (HDAC). Using cell lines with enforced expression of HBx, we demonstrated that HBx upregulated the expression of HMGB1 and promoted its Cyt translocation by acetylation to facilitate autophagy. We further identified the underlying mechanism by which decreased nuclear HDAC activity and expression levels contribute to the HBx‐promoted hyperacetylation and subsequent translocation of HMGB1. We also identified the HDAC1 isoform as a critical factor in regulating this phenomenon. In addition, HBx bound to HMGB1 in the cytoplasm, which triggered autophagy in hepatocytes. Pharmacological inhibition of HMGB1 Cyt translocation with ethyl pyruvate prevented HBx‐induced autophagy. These results demonstrate a novel function of acetylated HMGB1 in HBx‐mediated autophagy in hepatocytes.

**Abbreviations**
EP: ethyl pyruvate
HBV: hepatitis B virus
HBx: hepatitis B virus X
HCC: hepatocellular carcinoma
HDAC: histone deacetylase
HMGB1: high‐mobility group box 1
LC3: microtubule‐associated protein light chain 3
NSL1: nuclear localization signal 1
PPI: protein protein interaction
shRNA: small hairpin RNA
SP‐1: specificity protein 1
TSA: trichostatin A

## 1 Introduction

Hepatitis B virus (HBV) X protein (HBx) has been extensively studied as a critical protein in HBV infection and hepatocellular carcinoma (HCC) development. As a multifunctional viral protein, HBx functions in both the nucleus and cytoplasm. Nuclear HBx is involved in DNA activity‐associated events, such as DNA replication, repair, transcription, and genomic stability, whereas cytoplasmic (Cyt) HBx is involved in various cellular signal transduction pathways related to its transactivation (Levrero and Zucman‐Rossi, 2016; Na *et al*., 2016; Riviere *et al*., 2015). Previous studies showed that HBx could be involved in a complex network of epigenetic events that influence HBV infection and HCC development (Belloni *et al*., 2009; Pandey and Kumar, 2015; Yoo *et al*., 2008). Nuclear HBx was reported to recruit cellular histone acetyltransferases and histone deacetylases (HDACs) to modify the epigenetic regulation of cccDNA function. HBx was also shown to stabilize the SIRT7 deacetylase to promote cellular transformation (Pandey and Kumar, 2015). In addition, the acetylation/deacetylation of HIF‐1a by HBx may be a critical mechanism of HIF‐1a stabilization to facilitate liver cancer metastasis (Yoo *et al*., 2008). These studies suggested that cellular acetylation signaling could be a pivotal target utilized by the viral protein to exert its various functions. However, few data are available regarding the transcriptional regulation of HDACs by HBx and its involvement in the pathological process of HBV‐related diseases.

High‐mobility group box 1 (HMGB1) is a member of the HMG family, which plays a pivotal role in various forms of liver disease related to liver damage, fibrosis, and tumorigenesis (Chen *et al*., 2013). A wealth of evidence shows that HMGB1 is involved in the course of infections by viruses, including DNA and RNA viruses. HMGB1 protein was found to bind to influenza virus nucleoprotein and was required for the enhancement of influenza virus replication (Moisy *et al*., 2012). An interaction between HMGB1 and the phosphoprotein of Borna disease virus was shown to repress p53‐mediated transcription (Zhang *et al*., 2003). Moreover, HMGB1 was shown to be released from cells infected with a range of viruses, such as dengue virus, hepatitis C virus, and human immunodeficiency virus (Barqasho *et al*., 2010; Jung *et al*., 2011; Kamau *et al*., 2009). However, it remains largely unknown whether HMGB1 could be involved in the course of HBV infection through its interaction with the critical viral protein HBx. Recently, Chen *et al*. (2017) reported that HBx could trigger the secretion of HMGB1 via the CAMKK/CAMKIV pathway. Though the subcellular localization and function of HMGB1 are closely associated with its post‐translational modifications, it remains unclear whether and how HBx regulates the post‐translational modification of HMGB1.

Autophagy is a highly conserved and tightly regulated cellular process responsible for eliminating damaged organelles and proteins via a lysosomal pathway. Dysregulation of autophagy is associated with multiple liver diseases, including inflammation, virus infection, and cancer (Rautou *et al*., 2010). Our previous studies have identified Cyt HMGB1 as a critical pro‐autophagic protein that participates in autophagy by directly interacting with Beclin1 (Tang *et al*., 2010). However, it remains controversial whether HMGB1 is required for autophagy in liver diseases (Huang *et al*., 2014; Huebener *et al*., 2014). A growing body of evidence has revealed that HBV could exploit the autophagy machinery as a pivotal factor to favor viral replication via HBx (Sir *et al*., 2010). However, the underlying molecular mechanisms of HBx‐induced autophagy are only partially understood, and it is still unknown whether HMGB1 is involved in HBx‐mediated autophagy.

Considering the pivotal role of HBx and HMGB1 in the pathological process of liver diseases, we proposed that there might be a functional crosstalk between HBx and HMGB1. In this study, we aimed to determine the relationship between HBx and HMGB1, the mechanism and significance of HBx‐enhanced nucleo Cyt translocation of HMGB1, and the role of HMGB1 in HBx‐induced autophagy. Our findings define a new partner for HBx and identify an epigenetic mechanism (i.e., the induction of HMGB1 acetylation) by which HBx controls HBV‐mediated autophagy.

## 2 Materials and methods

### 2.1 Mice

The HBx mouse line was generated via homologous recombination using CRISPR/Cas9 technology by Shanghai Biomodel Organism Co., Ltd (Shanghai, China). All strains of mice used in this study were generated on a C57BL/6J background. Eight‐week‐old mice were used for all experiments. The mice were not fasted before sacrifice. Tissue harvesting was performed during the light cycle. This study was conducted in compliance with the regulations of the Institutional Animal Care and Use Committee of the State Key Laboratory of Medical Genetics of CSU in China.

### 2.2 Patients

Twenty‐four patients with HBV‐associated HCC who underwent tumor resection in Xiangya Hospital Central South University between March 2012 and May 2014 were included in the study. Samples of adjacent nontumor liver tissue were collected from these HCC patients. The patients were divided into high HBx and low HBx expression groups according to hepatic HBx mRNA levels. Written informed consent was obtained from all patients before performing liver surgery. This study was approved by the Ethics Committee of Xiangya Hospital Central South University (NO. 201509020).

### 2.3 Experimental approaches

1. Cell culture and reagents: Huh7 and HepG2 (hepatoma cell lines), L02 (an immortalized normal liver cell line), and HepG2.2.15 (a cell line containing the complete HBV genome) cells were purchased from the Cell Bank of the Chinese Academy of Sciences (Shanghai, China). Huh7, HepG2.2.15, and HepG2 cells were maintained in Dulbecco's modified Eagle's medium (DMEM; Gibco, Carlsbad, CA, USA), supplemented with 10% fetal bovine serum (FBS; Gibco), penicillin (100 U·mL^−1^), and streptomycin (100 μg°mL^−1^). L02 cells were maintained in RPMI medium (Gibco). The cells were incubated at 37 °C in a 5% CO_2_ incubator. Stable cell lines expressing HBx or lacking HMGB1 were constructed by lentivirus transduction. HMGB1 and specificity protein 1 (SP1) antibodies were obtained from Abcam (Cambridge, MA, USA). The HBx antibody was obtained from Xiamen University (Xiamen, Hujian, China). The HDAC2 antibody was obtained from Cell Signaling Technology (Danvers, MA, USA). The HDAC1 antibody was obtained from Santa Cruz Biotechnology (Dallas, TX, USA). The Flag antibody was obtained from Sigma‐Aldrich (Saint Louis, MO, USA). Tubulin, actin, and GAPDH antibodies were obtained from AURAGENE (Rockville, MD, USA). Acetylated lysine, P62, Beclin1, and LC3B antibodies were purchased from Novus Biologicals (Littleton, CO, USA). The HDAC activity assay kit was obtained from BioVision (Atlanta, GA, USA). The nuclear and Cyt extraction kit was obtained from Thermo Fisher Scientific (Rockford, IL, USA). Other reagents and kits were obtained from Sigma‐Aldrich (Auckland, New Zealand).
2. Plasmids: The Myc‐tagged full‐length HBx protein was constructed by inserting a PCR‐amplified full‐length HBx fragment into the Xhol/EcoRI sites of pcDNA3.1. The primer sequences used to amplify the Myc‐HBx plasmid were forward (5′‐ CCGCTCGAGATGGCTGCTAGGCTGTACTG‐3′) and reverse (5′‐ CCGGAATTCGGCAGAGGTGAAAAAGTTGC‐3′). Flag‐tagged HBx deletion mutants were constructed by inserting the ΔA–ΔE mutants of the human HBx cDNA to the XhoI/NheI sites of pcDNA3.1. The Flag‐tagged full‐length HMGB1 protein was constructed by inserting a PCR‐amplified full‐length HMGB1 fragment into the Xhol/EcoRI sites of pcDNA3.1. Flag‐tagged deletion mutants of HMGB1 were constructed by inserting the ΔA box, ΔB box, ΔC tail, before‐nuclear localization signal 1 (NSL1), NSL1, or after‐NSL1 mutants of the human HMGB1 cDNA into the HindIII/NotI sites of pcDNA3.1. The human HDAC1 promoter sequence containing SP1‐binding sites was cloned into the KpnI/HindIII sites of the pGL3‐Basic vector. The GFP‐LC3B plasmid was purchased from GenScript Biology. All new constructs were verified by DNA sequencing.
3. Gene transfection, RNAi, and luciferase reporter assays: Wild‐type (WT) or mutant HBx expression vectors and WT or mutant HMGB1 expression vectors were transfected into cells using Fugene6 reagent (0000181727; Promega, Madison, WI, USA) according to the manufacturer's instructions. Small interfering RNA (siRNA) duplexes against HBx (#1: sense: CCUUGAGGCAUACUUCAAATT; antisense: UUUGAAGUAUGCCUCAAGGTT, #2: sense: UCACCUCUGCACGUAGCAUTT; antisense: AUGCUACGUGCAGAGGUGATT) and negative control siRNA (Gene Pharma RNAi Company, Shanghai, China) were transfected into cells using transfection reagent (Lipofectamine 2000; Invitrogen, Auckland, New Zealand) according to the manufacturer's instructions. For the luciferase assay, cells were transiently cotransfected with the pRL‐null plasmid (Promega) containing the Renilla luciferase gene, which is used for internal normalization, and a construct containing the SP1‐binding region. Forty‐eight hours after transfection, cell lysates were prepared, and the luciferase activity was measured using a dual reporter assay system (Promega) according to the manufacturer's instructions. All transfections were performed in triplicate.
4. Western blotting: Whole‐cell lysates were separated by 6–15% SDS/PAGE, transferred to PVDF membranes (0.25 μm; Millipore, Billerica, MA, USA), and blocked for 1 h with 5% BSA and 0.1% Tween‐20 in TBS. The membranes were then washed and incubated separately with various primary antibodies. After incubation with peroxidase‐conjugated secondary antibodies for 1 h at 25 °C, the signals were visualized by enhanced chemiluminescence (Bio‐Rad, Hercules, CA, USA) according to the manufacturer's instructions. The relative band intensity was quantified using Image Lab software.
5. Immunoprecipitation (IP) analysis: Cells were lysed in RIPA buffer (50 mm Tris/Cl (pH 7.5), 150 mm NaCl, 1 mm EDTA, 0.1% SDS, 1% NP‐40, and 0.5% Na‐deoxycholate). The cell lysates were cleared by a brief centrifugation for 20 min at 12 000 ***g***. Before IP, samples containing equal amounts of proteins were precleared with protein A or protein G agarose at 4 °C for 4 h and subsequently incubated with 2–5 μg·mL^−1^ various specific antibodies or nonspecific IgG overnight at 4 °C with gentle shaking. Then, 20–80 μL of protein A or G agarose beads was added for 3 h. After incubation, the agarose/Sepharose beads were washed extensively with RIPA buffer, and proteins were eluted by boiling in 2× SDS sample buffer before SDS/PAGE.
6. RT‐PCR: Total RNA was extracted from cells with a Total RNA Kit II (Omega, Norcross, GA, USA). A total of 1 μg of RNA was reverse transcribed into cDNA using the cDNA Synthesis Kit (Takara, Otsu, Shiga, Japan). The PCR amplification conditions consisted of an initial denaturation at 95 °C for 3 min, followed by 40 cycles of 95 °C for 30 s, 60 °C for 30 s, and 95 °C for 15 s.
7. ChIP assay: ChIP was performed using a ChIP‐IT Express kit (Active Motif, Carlsbad, CA, USA). For quantitative ChIP assays, real‐time PCR was performed with an RT Real‐TimeTM SYBR Green/ROX PCR Master Mix (Bio‐Rad) according to the supplier's instructions. The HDAC1 promoter region, −106 to +78 bp, containing SP1‐binding sites, was PCR‐amplified using purified DNA as a template. The primer sequences used to amplify the region −106 to +78 were forward (5′‐TGGCTGGAGCGGTGCCC‐3′) and reverse (5′‐ GGCTCCGCTCAGCGTCCG‐3′).
8. Autophagy assays: All assays to detect endogenous microtubule‐associated protein light chain 3 (LC3) puncta were performed by imaging cytometry as previously described (Tang *et al*., 2010). Autophagic vesicle formation was further monitored by transient expression of GFP‐LC3B aggregation in cells. Autophagy assays were performed by western blotting for LC3‐I/II formation after transfection with HBx in the presence or absence of HMGB1. Transmission electron microscopy assessment of autophagosomes and autophagolysosomes was performed as previously described (Tang *et al*., 2010).
9. Measurement of HDAC activity: First, 200 μg of cell lysate was diluted with 85 μL of ddH_2_O in each well of an analysis plate. Then, 10 μL of 10× HDAC assay buffer was added to each well, followed by 5 μL of HDAC colorimetric substrate, and the solutions were thoroughly mixed. The plate was incubated at 37 °C for 1 h. The reaction was stopped by adding 10 μL of lysine developer and thoroughly mixing, and the plate was incubated at 37 °C for 30 min. The samples were read in an ELISA plate reader at 405 nm.

### 2.4 Statistical analysis

Data are expressed as the mean ± SD of three independent experiments performed in triplicate. Student's *t*‐test was used to test continuous variables with normal distribution. The correlations between groups were analyzed using Pearson's correlation test. Statistical significance was defined as a *P*‐value of < 0.05.

## 3 Results

### 3.1 HBx upregulates HMGB1 expression and promotes its cytoplasmic translocation

**Figure 1:**
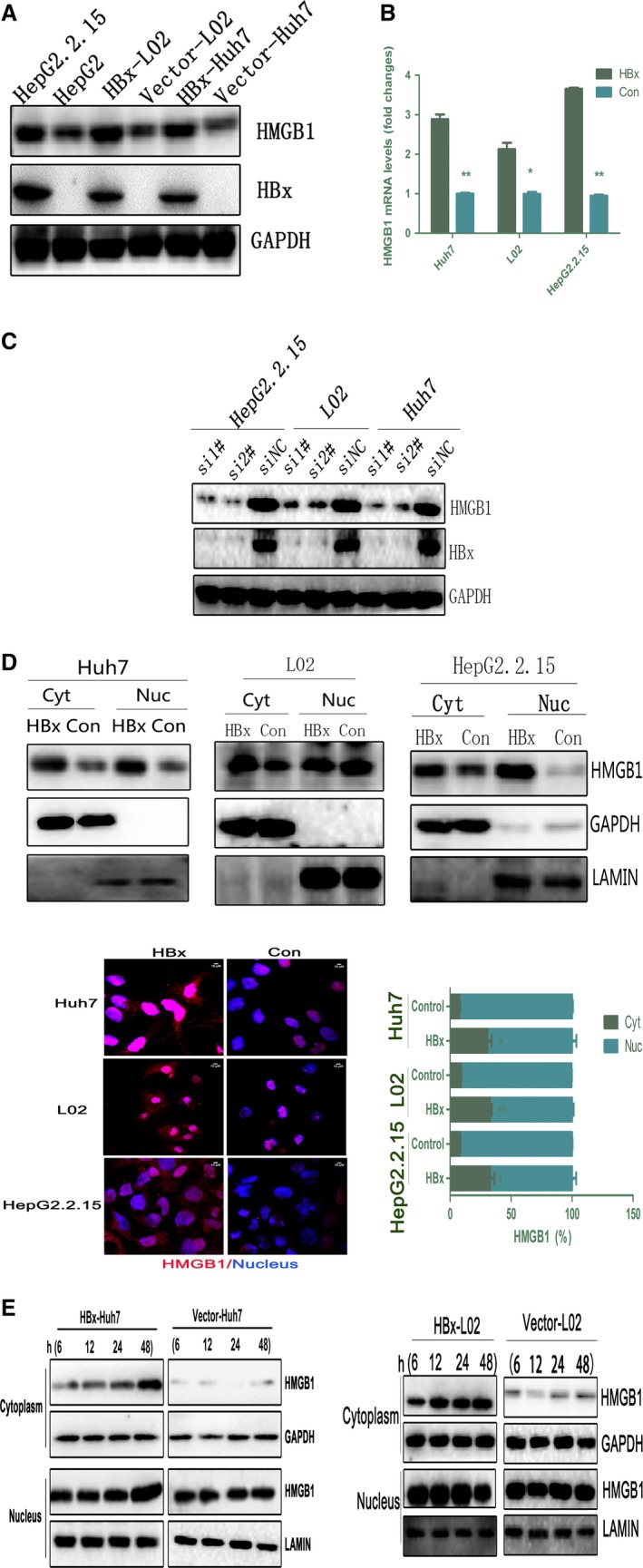
HBx upregulates HMGB1 expression and promotes its Cyt translocation. (A,B) HMGB1 expression levels in HepG2.2.15, HBx‐Huh7, and HBx‐L02 cells were analyzed by immunoblot and RT‐PCR. *P <* *0.05, **P <* *0.01 compared with empty vector, *n* = 3. (C) The indicated cells were pretreated with HBx‐specific siRNA for 72 h and immunoblotted with an HMGB1‐specific antibody. (D) Nuclear/Cyt HMGB1 expression in cells stably transfected with HBx (HBx‐Huh7, HBx‐L02, and HepG2.2.15) was analyzed by immunoblot and immunofluorescence. LAMIN was used as a nuclear fraction control, and GAPDH was used as a Cyt fraction control. The mean nuclear (Nuc) and Cyt HMGB1 intensity per cell was determined by immunofluorescence. *P <* *0.05, **P <* *0.01 versus the empty vector (Control) group; *n *=* *3. Representative images are depicted on the left, HMGB1 (red) and DAPI (blue). Scale bar, 10 μm. (E) Immunoblot analysis of nuclear/Cyt HMGB1 expression in cells (Huh7 and L02) transiently transfected with HBx (3 μg) for the indicated time periods (6, 12, 24, and 48 h).

We first analyzed the expression profiles of HMGB1 in HBx‐expressing cells. As shown in Fig. 1A,B, both the HMGB1 mRNA and protein levels were significantly increased in cells expressing HBx (HepG2.2.15, HBx‐L02, and HBx‐Huh7) compared with cells transfected with empty vector. Most strikingly, immunoblotting analysis for both HBx and HMGB1 in L02 cells transfected with HBx for different time periods (36–72 h) indicated that HBx levels peaked at 72 h, concomitant with notable HMGB1 upregulation (Fig. S1). We then performed immunoblotting analysis of HMGB1 after HBx knockdown via siRNA treatment, and a notable reduction in HMGB1 protein levels was observed (Fig. 1C).

We next examined the subcellular distribution of HMGB1 in cells stably transfected with HBx. Interestingly, HBx overexpression obviously promoted HMGB1 Cyt translocation, as assessed by western blot analysis of subcellular fractions and cytometry imaging (Fig. 1D). Furthermore, in both Huh7 and L02 cells, transient transfection with HBx for various time periods promoted HMGB1 translocation, which peaked at 48 h, compared with cells transfected with empty vector (Fig. 1E). These results confirmed that HBx upregulated HMGB1 expression at the transcriptional level and induced its translocation in a time‐dependent manner, without decreasing cell viability (Fig. S2).

### 3.2 HBx induces HMGB1 acetylation by regulating HDAC activity and expression

**Figure 2:**
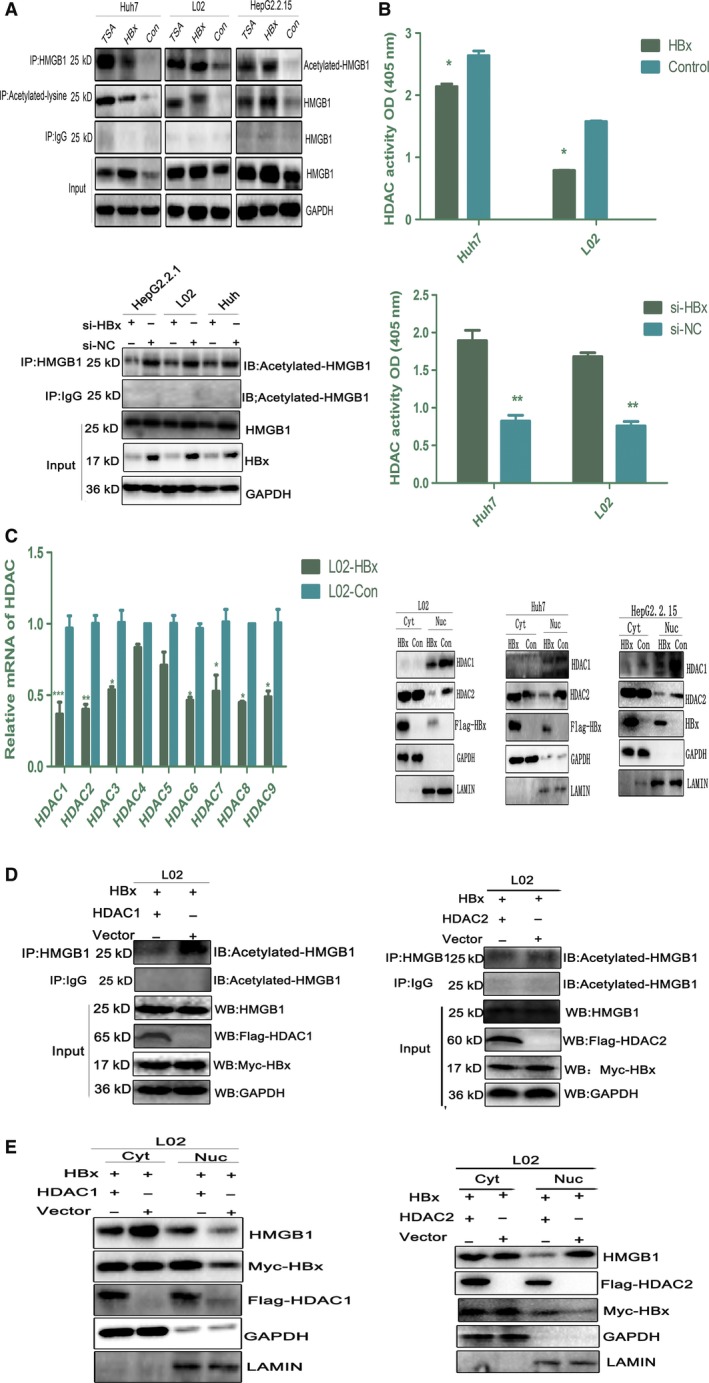
HBx induces HMGB1 acetylation by regulating HDAC activity and expression. (A) HBx promotes the acetylation of HMGB1. TSA (1 μm, 24 h) treatment was used as a positive control. Whole lysates of HBx‐L02, HBx‐Huh7, and HepG2.2.15 cells were immunoprecipitated with an acetylated lysine or HMGB1 antibody and then subjected to immunoblot analysis with the indicated antibodies. Samples were pulled down with anti‐HMGB1 and immunoblotted with anti‐acetylated lysine following HBx knockdown via siRNA treatment for 72 h. (B) HDAC activity was determined by colorimetric assay after HBx overexpression or knockdown. *P <* *0.05, **P <* *0.01, *n* = 3. (C) RT‐PCR analysis of HDAC1–9 expression in HBx‐L02 and Vector‐L02 cells. *P <* *0.05, **P <* *0.01, *n* = 3. Immunoblot analysis of nuclear/Cyt HDAC1 or HDAC2 expression in HBx‐L02 and Vector‐L02 cells. (D and E) L02 cells were cotransfected with Myc‐tagged HBx (1.5 μg) and Flag‐tagged HDAC1 (1.5 μg) or Flag‐tagged HDAC2 (1.5 μg) for 48 h. Whole lysates were immunoprecipitated with anti‐HMGB1 and immunoblotted with anti‐acetylated lysine. Nuclear/Cyt HMGB1 distribution was then analyzed by western blot.

Previous studies have suggested that the mobilization of nuclear HMGB1 is mediated by post‐translational modifications, and acetylation appears to be critical for active HMGB1 translocation. HBx has been reported to recruit its partners CBP, p300, and PCAF to contribute to nuclear histone acetylation (Riviere *et al*., 2015). We next explored whether HBx could acetylate nuclear HMGB1 in HBx‐Huh7, HBx‐L02, and HepG2.2.15 cells. As shown in Fig. 2A, treatment with the class I HDAC inhibitor trichostatin A (TSA) or HBx overexpression resulted in an evident increase in acetylated HMGB1. Conversely, HBx knockdown partly abrogated HMGB1 acetylation (Fig. 2A). These results suggested that HBx regulates HMGB1 acetylation, thereby promoting its Cyt translocation.

To evaluate whether the HBx‐mediated increase in HMGB1 acetylation was related to the suppression of HDAC activity, we first examined the HDAC activity in HBx‐L02 and HBx‐Huh7 cells. As expected, HBx overexpression resulted in a notable downregulation of HDAC activity. Moreover, HBx blockade obviously reversed this effect (Fig. 2B). Next, we determined the expression levels of the nine HDAC subtypes expressed in HBx‐L02 cells and found that most subtypes were downregulated by HBx at the mRNA level, especially *HDAC1* and *HDAC2* (Fig. 2C). Since HDAC activities are associated with their intracellular locations, we next analyzed the nuclear/ Cyt distribution of HDAC1 and HDAC2 in the context of HBx overexpression. Intriguingly, we found that HBx obviously downregulated HDAC1 expression in the nucleus but had no effect on its nuclear translocation. However, the nuclear translocation of HDAC2 was greatly impaired in HBx‐L02 cells compared with controls cells transfected with empty vector (Fig. 2C). This result suggested that HBx‐mediated HMGB1 acetylation at least partly resulted from the downregulation of *HDAC1* and *HDAC2* transcriptional expression and the consequent suppression of HDAC activity. However, we could not rule out the participation of other mechanisms in this process, such as augmented p300/PCAF levels and the inactivation of class II HDACs. Therefore, we further evaluated the expression of CBP/p300 and PCAF in HBx‐L02 cells. As shown in Fig. S3, both the mRNA and proteins levels of CBP/p300 and PCAF were unaltered in cells (Huh7 and L02) transfected with HBx compared with control cells transfected with empty vector. These data strongly support that the downregulation of HDAC activity and transcriptional expression are important mechanisms in HBx‐mediated HMGB1 acetylation.

Next, to further characterize the crosstalk between HDACs and HMGB1 nucleocytoplasmic shuttling in the setting of enhanced HBx expression, we evaluated the acetylation status and subcellular localization of HMGB1 in HBx‐L02 cells overexpressing HDAC. We found that HDAC1 overexpression obviously abrogated HBx‐mediated HMGB1 acetylation, concomitant with a notable reduction in HMGB1 Cyt translocation. By contrast, HDAC2 overexpression had no effect on either HMGB1 acetylation or its subsequent Cyt shuttling (Fig. 2D,E). Moreover, when cells (Huh7, L02, and HepG2.2.15) were treated with TSA, a significant increase in HMGB1 acetylation was observed. A dose of 1 μm TSA efficiently induced HMGB1 Cyt translocation without toxicity to the cells at up to 24 h following treatment (Fig. S4A,B). These results showed that HDAC inhibition by HBx promoted the acetylation and nuclear‐to‐Cyt translocation of HMGB1 and that the isoform HDAC1 is a critical regulator of HBx‐induced acetylation of HMGB1.

### 3.3 HBx regulates HDAC1 expression via the transcription factor‐specific protein 1 (SP1)

**Figure 3:**
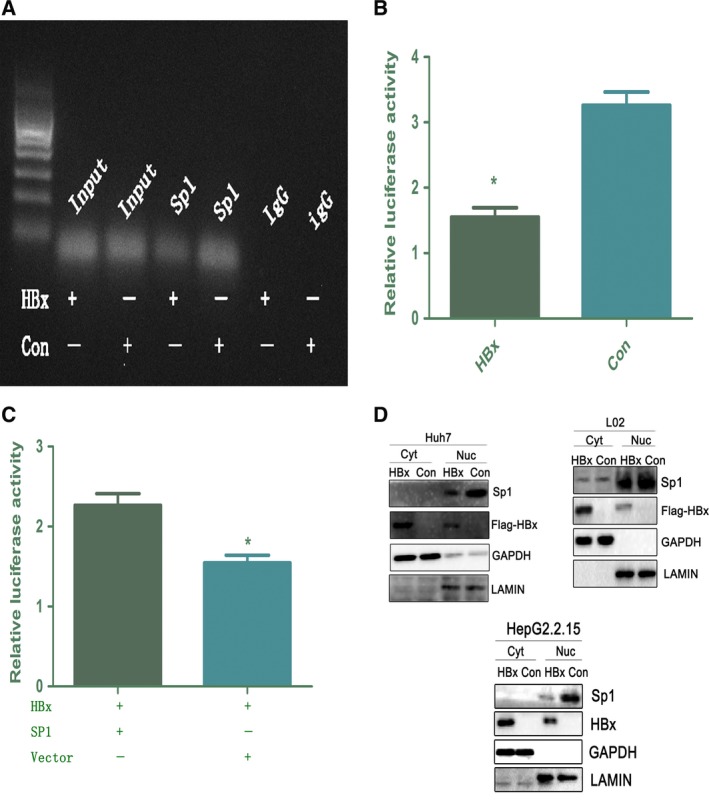
HBx regulates HDAC1 expression via SP1. (A). A ChIP assay demonstrates a decrease in SP1 binding to the HDAC1 promoter following HBx overexpression. (B,C). 293Tcells were transfected with reporter constructs derived from the pGL3‐Basic vector containing SP1‐binding sites in the promoter of human HDAC1 gene as indicated. *P <* *0.05, *n* = 3. HDAC1 promoter activity was reversed after cotransfection with HBx (1.5 μg) and SP1 (1.5 μg). (D). Immunoblot analysis of the subcellular distribution of SP1 in HBx‐Huh7, HBx‐L02, and HepG2.2.15 cells.

Microarray analysis showed that the activity of the transcription factor SP1 was significantly downregulated in HBx‐L02 cells (data not shown). As the *HDAC1* promoter contains several putative SP1‐binding sites and SP1 was reported to be a potent transactivator of the mouse *Hdac1* promoter (Schuettengruber *et al*., 2003), we speculated that the downregulation of human *HDAC1* expression at the transcriptional level by HBx occurs via inhibition of SP1 activity. To verify this speculation, we first evaluated whether SP1 was indeed associated with the human *HDAC1* promoter *in vitro*. As shown in Fig. 3A, a ChIP assay revealed that HBx overexpression in L02 cells could significantly weaken the binding of SP1 to the *HDAC1* promoter region. Next, we performed a dual‐luciferase reporter assay in 293T cells transfected with HBx. As shown in Fig. 3B, HBx transfection significantly decreased the activity of the human *HDAC1* promoter containing SP1‐binding sites. Conversely, SP1 overexpression obviously reversed this effect (Fig. 3C). These findings suggest that modulation of SP1 activity is crucial for human *HDAC1* promoter activity. Indeed, as shown in Fig. 3D, HBx overexpression could significantly downregulate the expression of SP1 but had no impact on its nuclear translocation. These data suggested that HBx contributes to *HDAC1* transcription inhibition in an SP1‐dependent manner.

### 3.4 HBx binds HMGB1 in the cytoplasm

**Figure 4:**
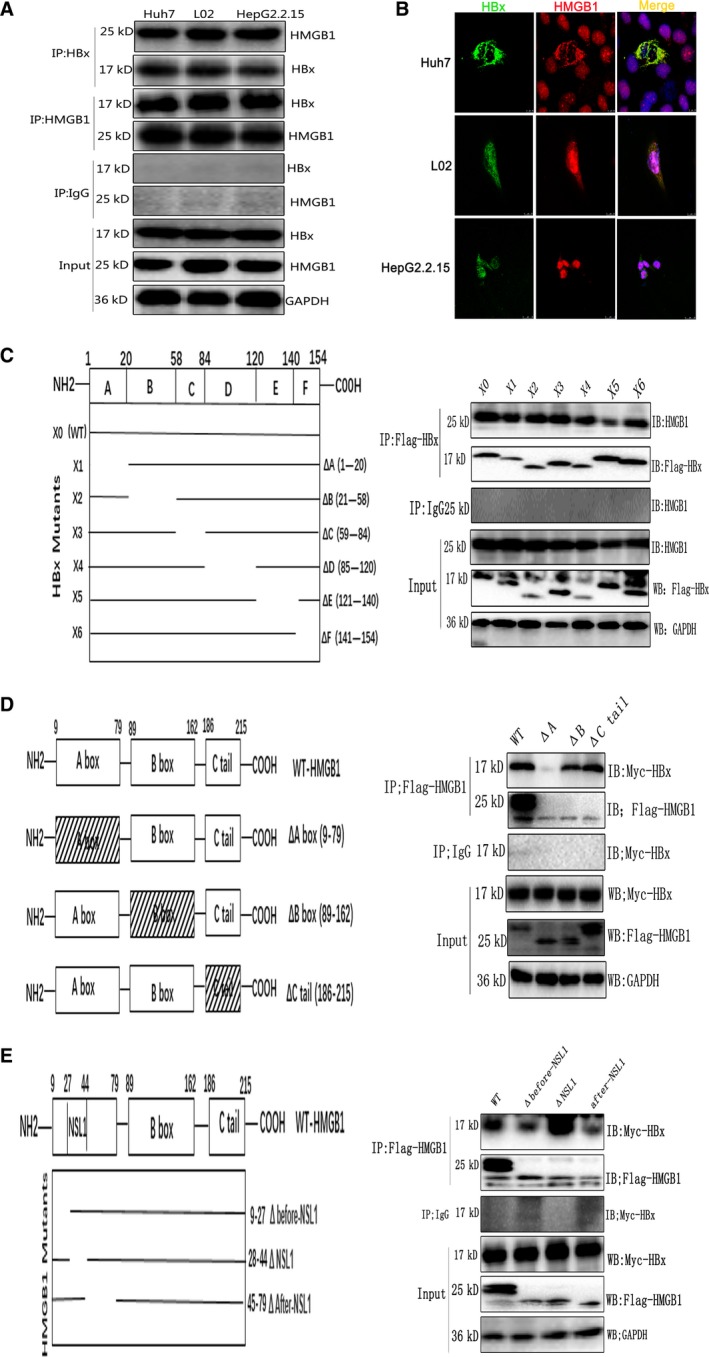
HBx binds HMGB1 in the cytoplasm. (A). Whole‐cell extracts of HBx‐Huh7, HBx‐L02, and HepG2.2.15 cells were immunoprecipitated with the indicated antibodies, and the immunoprecipitates were analyzed by immunoblotting. (B) Colocalization of HBx and HMGB1 was analyzed by confocal microscopy. (C) Scheme of HBx mutants. The 154‐amino‐acid WT HBx sequence (X0) was divided into six regions (A–F). The line indicates the portion of HBx present in the mutant, with the deleted region shown as a gap. 293T cells were transfected with Flag‐tagged full‐length HBx and deletion (Δ) mutants (X1–X6) of HBx. Cell lysates were immunoprecipitated with anti‐Flag and probed by western blot with anti‐HMGB1. (D) Scheme of HMGB1 mutants. The 215‐amino‐acid WT HMGB1 sequence was divided into three regions, including the A box, B box, and C‐tail. The box with hatch marks indicates the deleted region of HMGB1. 293T cells were transfected with Myc‐HBx and Flag‐tagged WT HMGB1 or the A box, B box, or C‐tail deletion of HMGB1. Cell lysates were immunoprecipitated with anti‐Flag and probed by western blot with anti‐Myc. (E) The 70‐amino‐acid A box in HMGB1 was further divided into three regions based on the location of NSL1, that is, one NSL1‐containing domain, one domain before NSL1 and one domain after NSL1. The line indicates the portion of HMGB1 present in the mutant, with the deleted region shown as a gap. 293T cells were transfected with Myc‐HBx and Flag‐tagged WT HMGB1 or the NSL1, before‐NSL1, or after‐NSL1 deletion of HMGB1. Cell lysates were immunoprecipitated with anti‐Flag and probed by western blot with anti‐Myc.

Since Cyt HMGB1 is a pivotal regulator of cellular autophagy, and HBx is thought to play a crucial role in HBV‐induced autophagy, we speculated that HBx‐induced autophagy is dependent on HMGB1. To test this hypothesis, we first investigated whether HBx interacts with HMGB1 using IP assays. As shown in Fig. 4A, HBx and HMGB1 coimmunoprecipitated reciprocally in cells expressing HBx (HBx‐Huh7, HBx‐L02, and HepG2.2.15). Because both HBx and HMGB1 have various intracellular locations, we aimed to determine where the two proteins might specifically interact with each other. Confocal microscopy revealed that HBx and HMGB1 clearly colocalized in the cytoplasm (Fig. 4B). Next, we sought to determine which protein regions are responsible for the interaction between HBx and HMGB1. First, six HBx polypeptide deletion mutants were made. In particular, the X5 deletion mutant, which lacked the E domain, was identified as the HMGB1‐binding site(Fig. 4C). Deletion mapping analysis demonstrated that the A box domain of HMGB1 bound strongly to HBx (Fig. 4D). Since the A box contains a NLS1, we then further searched for the precise interacting sites of HBx in the A box of HMGB1. As shown in Fig. 4E, deletion of the region after‐NSL1 domain in the A box resulted in a notable reduction in the binding between HBx and HMGB1. Together, these data suggest that HBx interacts with HMGB1 in the cytoplasm via binding of the after‐NSL1 domain in HMGB1 and the E domain in HBx.

### 3.5 HBx induces autophagy in an HMGB1‐dependent manner

**Figure 5:**
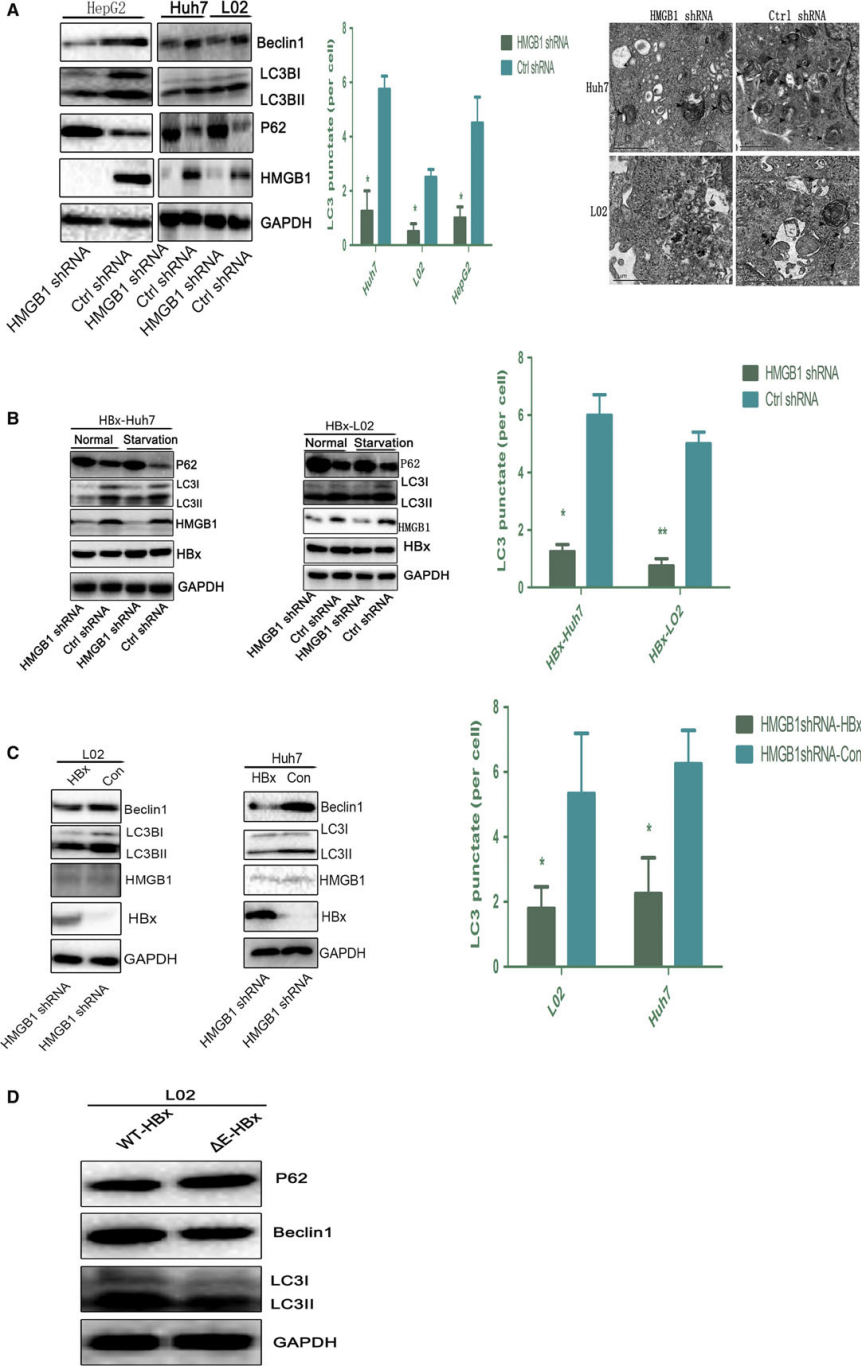
HBx induces autophagy in an HMGB1‐dependent manner. (A) The expression of the indicated proteins and LC3 puncta was detected in cells (Huh7, L02, and HepG2) transfected with HMGB1 shRNA or Ctrl shRNA by immunoblot and immunofluorescence, respectively. *P <* *0.05, **P <* *0.01, *n* = 3. Ultrastructural features in the indicated cells were observed by electron microscopy. (B) Immunoblot detection of the indicated proteins in cells treated with HMGB1 shRNA and Ctrl shRNA and transfected with HBx under basal conditions and in response to starvation. LC3 puncta formation was assayed by immunofluorescence under basal conditions, *P <* *0.05, **P <* *0.01, *n* = 3. (C) Western blot analysis of the indicated proteins in cells (L02 and Huh7) treated with HMGB1 shRNA and transfected with HBx or empty vector for 48 h. LC3 puncta formation was assayed by immunofluorescence. *P <* *0.05, *n* = 3. (D) The indicated proteins were analyzed by immunoblot in L02 cells transfected with WT HBx (3 μg) and an HBx mutant with deletion of the E domain (3 μg).

We next determined whether HBx could trigger autophagy. Cells expressing HBx showed upregulation of Beclin1 protein levels, enhanced accumulation of light chain 3 protein‐II (LC3II), and reduction in SQSTM1/P62 levels. Microscopy analysis of GFP‐tagged LC3B revealed that HBx increased the number of GFP‐LC3B puncta compared with that of control cells. Moreover, ultrastructural analysis indicated that cells transfected with HBx exhibited a larger number of autophagic vacuoles per cell than control cells (Fig. S5A). By contrast, knockdown of HBx expression obviously abrogated this autophagic effect (Fig. S5B), suggesting a pivotal role of HBx in autophagy induction. Next, we evaluated autophagic activity in cells (HepG2, Huh7, and L02) transfected with a small hairpin RNA (shRNA) targeting *HMGB1*. As shown in Fig. 5A, these cells had markedly diminished Beclin1 protein levels, LC3‐II expression, GFP‐LC3 puncta levels, and autophagosome formation, indicating that HMGB1 is indeed an important factor regulating autophagy in hepatocytes and hepatoma cells. To evaluate the role of HMGB1 in HBx‐induced autophagy, we conducted an immunoblotting analysis of autophagy in cells (L02 and Huh7) treated with *HMGB1* shRNA and overexpressing HBx under basal conditions and in response to starvation. As expected, a lack of HMGB1 limited autophagy in cells transfected with HBx in both basal and starvation conditions, as shown by decreased levels of LC3‐II and accumulation of GFP‐LC3B puncta and increased levels of P62 (Fig. 5B) relative to cells transfected with control shRNA. These results demonstrated that the function of HBx in autophagy induction was HMGB1‐dependent. Next, to determine whether other molecular mechanisms were involved in HBx‐mediated autophagy, we investigated the autophagy activity in cells expressing HBx in the context of HMGB1 depletion. Importantly, cells (L02 and Huh7) treated with *HMGB1* shRNA cells and transiently expressing HBx showed a remarkable decrease in LC3‐II expression, Beclin1 levels, and GFP‐LC3 puncta compared with empty vector‐transfected cells (Fig. 5C), suggesting an indispensable role of HMGB1 in HBx‐triggered autophagy. Finally, to further determine the role of the HBx–HMGB1 complex in regulating autophagy *in vitro*, we evaluated autophagy in L02 cells transfected with WT HBx and an HBx mutant with alteration of the binding domain for HMGB1. Notably, a significant abrogation in autophagy was observed when region E was deleted (Fig. 5D). Thus, we concluded that region E of HBx is critical for the HBx–HMGB1 interaction and HBx‐mediated autophagy.

### 3.6 Cytoplasmic HMGB1 is required for HBx‐induced autophagy

**Figure 6:**
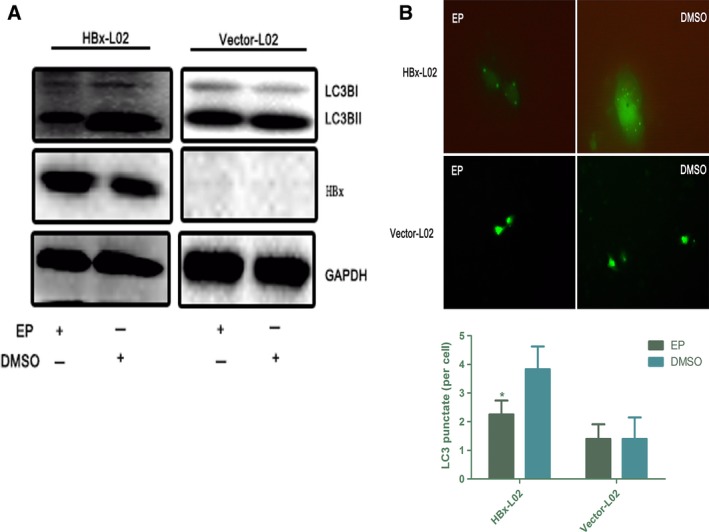
Cyt HMGB1 is required for HBx‐induced autophagy. (A,B) Analysis of LC3 processing by immunoblot and immunofluorescence in HBx‐L02 and Vector‐L02 cells after EP or DMSO (0.25 mm, 24 h) treatment. Quantitative GFP‐LC3 data are shown on the right. *P <* *0.05, *n* = 3.

Ethyl pyruvate (EP) is a reactive oxygen species scavenger and inflammatory suppressor. Treatment with EP has been shown to inhibit HMGB1 Cyt translocation and extracellular release (Ulloa *et al*., 2002). To further confirm that Cyt HMGB1 regulates the autophagy triggered by HBx, we treated HBx‐L02 cells with various concentrations of EP for 24 h. As shown in Fig. S6, 0.25 mm EP could efficiently abrogate HMGB1 Cyt translocation. Next, we performed immunoblotting and microscopic analysis of autophagy in HBx‐L02 cells treated with the indicated concentrations of EP for 24 h. As expected, EP treatment efficiently blocked the HBx‐induced aggregation of GFP‐LC3 puncta and LC3‐II expression, whereas no such effects were observed in Vector‐L02 cells (Fig. 6A,B). These results are consistent with the notion that HMGB1 Cyt translocation is required for HBx‐mediated autophagy.

Our previous study showed that Cyt HMGB1 interacts with Beclin1, thereby promoting autophagy (Tang *et al*., 2010). To investigate the mechanism(s) by which HBx regulates this autophagic activity, we analyzed the interaction between Beclin1 and HMGB1 in cells expressing HBx. As expected, formation of the HMGB1/Beclin1 complex was increased in HepG2.2.15 and HBx‐L02 cells compared with cells transfected with empty vector, as assessed by immunoblot analysis of subcellular fractions. Moreover, the colocalization of HMGB1 and Beclin1 was further observed in the cytoplasm in liver samples from HBV patients when examined by confocal microscopy (Fig. S7A,B).

### 3.7 Interaction of HBx and HMGB1 *in vivo*

**Figure 7:**
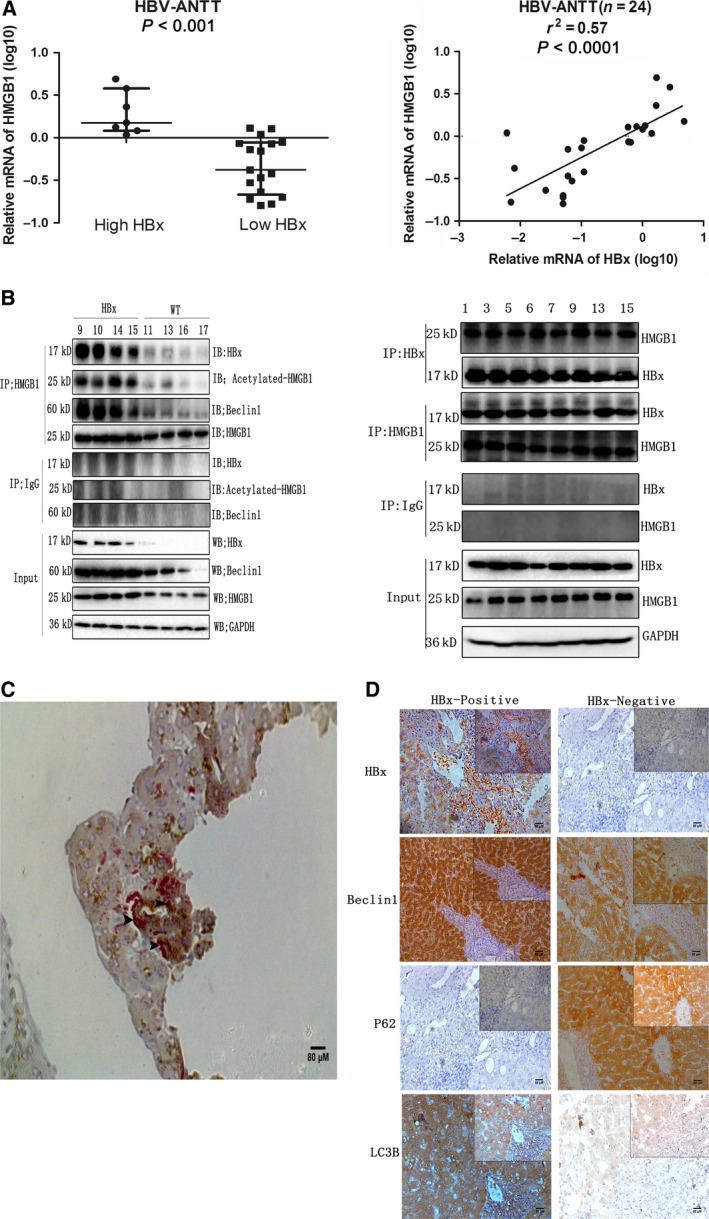
Interaction of HBx and HMGB1 *in vivo*. (A) mRNA levels of intrahepatic HBx and HMGB1 were examined by RT‐PCR, and the correlation between HBx and HMGB1 was assessed by linear regression (*r* ^2^ = 0.57, *P *<* *0.0001). ANTT: Adjacent nontumor tissue. (B) HBx/HMGB1, HMGB1/Beclin1, and HMGB1/acetylated lysine complexes immunoprecipitated from liver samples from HBx‐transgenic mice or HBV patients. (C,D) Double‐stained liver tissue sections from HBV patients showing HBx/HMGB1 expression and colocalization. Brown: HBx, Red: HMGB1. Arrows indicate the overlap of HBx and HMGB1. Immunohistochemical analysis of the indicated proteins in the livers of patients in the HBx‐positive and HBx‐negative expression groups.

To further characterize the crosstalk between HBx and HMGB1 *in vivo*, we analyzed the mRNA levels of *HMGB1* in liver samples from HBV patients. As expected, the HBx‐high expression group had much higher *HMGB1* mRNA levels than the HBx‐low expression group, and there was a positive correlation between the intrahepatic HBx and HMGB1 mRNA levels as assessed by RT‐PCR (Fig. 7A). Next, we examined the subcellular localization and acetylation of HMGB1 in liver samples with HBx expression, and clear Cyt localization and acetylation of HMGB1 were observed (Fig. 7B,C). Finally, we examined the HBx–HMGB1 and HMGB1–Beclin1 interactions in liver samples. Intriguingly, formation of these complexes was increased in liver samples from both HBx‐transgenic mice and HBV patients when examined by co‐IP assays (Fig. 7B). Moreover, colocalization of HBx and HMGB1 in the cytoplasm was further confirmed by a double‐staining assay in HBV patients (Fig. 7C). Notably, the hepatic protein levels of LC3‐II and Beclin1 were obviously higher in liver samples from HBx‐transgenic mice and HBV patients than in control liver samples, whereas the P62 levels were markedly diminished, as assessed by immunoblot and immunohistochemical staining (Fig. 7D and Fig. S8B). Together, these results strongly support our hypothesis that the crosstalk between HBx and HMGB1 facilitates autophagy in the liver.

## 4 Discussion

Hepatitis B virus X and HMGB1 are important proteins involved in the pathological processes of HBV‐related diseases. HBx acts as an oncoprotein, promoting HBV infection and HCC development mainly by interacting with host proteins, while HMGB1 plays a critical role in cell survival via autophagy induction. Previous studies have suggested that HMGB1 interacts with viral proteins to participate in the course of infection by viruses such as influenza and Borna disease (Moisy *et al*., 2012; Zhang *et al*., 2003). Here, we provide the first evidence that HMGB1 also interacts with the critical viral protein HBx and that this binding facilitates autophagy in hepatocytes and hepatoma cells. Previous studies have indicated that acetylation is a pivotal regulator that controls the subcellular localization of HMGB1 (Bonaldi *et al*., 2003). In the present study, we demonstrated that HBx could induce the active Cyt shuttling of HMGB1, which was related to its increased acetylation and the suppression of HDAC enzymatic activity. Our study showed for the first time that HMGB1 acetylation was associated with HBx function. This suggests that there might be various host proteins whose acetylation status is regulated by HBx and that HBx‐mediated epigenetic changes could participate in a wide range of biological processes. Thus, understanding the epigenetic events mediated by HBx would definitely provide new insights into HBV infection and HCC development.

Histone deacetylasea are members of an ancient enzyme family that has recently received increased attention. To deacetylate host proteins, HDACs need to localize in the nucleus, where their predominant substrate is found (Sengupta and Seto, 2004). Early studies of human HDAC1 and HDAC2 revealed that the two isoforms coexist as multiprotein complexes in the nucleus to exert their effects (Humphrey *et al*., 2001). Here, we found that transfection with HBx severely impaired HDAC2 nuclear translocation, whereas that of HDAC1 was not affected. Moreover, diffuse Cyt localization of HDAC2 was discovered in liver samples from HBV patients (data not shown). Thus, the prevention of HDAC2 nuclear translocation together with the disruption of the HDAC1–HDAC2 complex by HBx may account for the observed decrease in HDAC enzymatic activity. In addition, we found that *HDAC1* overexpression could efficiently abrogate HBx‐mediated HMGB1 acetylation and subsequent Cyt translocation. Thus, we identified the *HDAC1* isoform as a critical factor in regulating HBx‐mediated HMGB1 acetylation. Conversely, inhibition of HDAC1 by TSA notably reversed this effect. Overall, these findings suggest a pivotal role for HDACs in regulating HBx‐induced HMGB1 acetylation. However, the means by which HDACs themselves are regulated are poorly understood. A previous study showed that HBx could induce HDAC1 expression at the transcriptional level to enhance hypoxia signaling in hepatoma cells (Yoo *et al*., 2008). By contrast, our results suggest that HBx obviously downregulated HDAC1 mRNA expression *in vitro* and that SP1 is the key transcription factor that fails to bind to the *HDAC1* promoter, thereby suppressing its expression. HDACs play a critical role in the deacetylation of host proteins, thereby silencing gene expression. In our study, the inhibition of HDACs by HBx may account for the wide transactivation activities of HBx. Gaining a better understanding of the regulation of the expression of *HDACs* as well as their enzymatic activity, post‐transcriptional modifications and subcellular distribution can help elucidate the mechanisms contributing to host protein acetylation. Our findings point to HDAC1 as the key isoform associated with HMGB1 acetylation and Cyt translocation. However, other HDACs and histone acetyltransferases may also participate in HMGB1 acetylation, and we aim to continue to investigate these mechanisms in the near future.

Autophagy is an intracellular degradation pathway that targets Cyt components for lysosomal degradation. HBV has been shown to exploit the host autophagy mechanisms for its own replication via HBx (Sir *et al*., 2010). However, it remains unclear whether the positive autophagy regulator HMGB1 is functionally relevant in HBx‐induced autophagy. In this study, our data showed that HBx could upregulate the expression of *HMGB1* at the transcriptional level both *in vitro* and *in vivo*. Moreover, HBx also interacted with HMGB1, and our data support an indispensable role for HMGB1 in HBx‐mediated autophagy. Indeed, HBx overexpression induced autophagic activity, and its blockade led to autophagy impairment, suggesting that HBx is an important autophagy inducer. However, HMGB1 knockdown significantly abrogated the autophagy triggered by HBx, suggesting that HBx‐induced autophagy is HMGB1‐dependent. A recent study from Schwabe's laboratory indicated that HMGB1 is not required for autophagy in the liver (Huebener *et al*., 2014). By contrast, our results indicate that HMGB1 is indeed an important factor regulating autophagy in the liver, given that HMGB1 depletion could efficiently limit autophagy in L02, Huh7, and HepG2 cells. Moreover, we demonstrated that HMGB1 translocation in the setting of enhanced HBx expression served as a signal to initiate autophagy, since inhibition of HMGB1 Cyt translocation by EP efficiently limited HBx‐induced autophagy. Cyt HMGB1 then participates in autophagy by interacting with Beclin1, a process that is regulated by HBx. Thus, via regulating HMGB1 expression and its subcellular localization, HBx contributes to Beclin1–HMGB1 complex formation to facilitate autophagy.

Based on the present work, we propose a model of how HBx controls autophagy in an epigenetic manner. In this model, HBx generated by HBV infection induces HMGB1 acetylation and its subsequent Cyt translocation via HDAC inhibition. HDACs tilt the acetylation/deacetylation balance of HMGB1 in response to HBx. Our data showed for the first time that a positive crosstalk exists between HBx and HMGB1 *in vivo and vitro* and that the induction of autophagy by HBx is dependent on HMGB1. As HMGB1 dysfunction has been implicated in various forms of liver disease, ranging from liver damage to fibrosis, as well as in tumorigenesis, our novel findings further elucidate the precise role of HMGB1 in HBV‐related diseases. In addition to autophagy, the crosstalk between HBx and HMGB1 may also participate in other cellular biological behaviors, such as proliferation, apoptosis, invasion, and metastasis. Therefore, extensive research is still needed to determine the relationship between the viral protein HBx, the host protein HMGB1, and liver diseases. Understanding the crosstalk between HBx and HMGB1 in more detail will provide better insight into HBx‐mediated biological functions and will guide therapeutic intervention in liver diseases.

## Author contributions

X‐GF, NL, DT, and LS conceived and designed the project. SF, JW, YF, and XH acquired the data. SF, RZ, RK, and YH analyzed and interpreted the data. SF and NL wrote the manuscript.

## Conflict of interest statement

We declare that we have no financial or personal relationships with other people or organizations that could inappropriately influence our work and that there is no professional or other personal interest of any nature or kind in any product, service and/or company that could be construed as influencing the position presented in, or the review of, the manuscript entitled, ‘Crosstalk between HBx and HMGB1Facilitates Autophagy in Hepatocytes’.

## Supporting information


**Fig. S1.** HMGB1 expression at the indicated time points after transfection with HBx(3 μg)in L02 cells was analyzed by Western blot.

**Fig. S2.** Cell viability was determined by CCK8 assay after cells (Huh7 and L02 )transfected with HBx(3 μg)at indicated time periods(48 h).

**Fig. S3.** Expression levels of CBP/P300 and PCAF in HBx‐L02 and Vector‐L02 cells were detected by RT‐PCR and Western blot.

**Fig. S4.** HDAC inhibition by TSA promotes the Cyt translocation of HMGB1.

**Fig. S5.** HBx triggers autophagy in hepatocytes.

**Fig. S6.** HBx‐L02 cells were treated with EP (0.1–0.5 mm) for 24 h.

**Fig. S7. (A)** HBx overexpression promotes HMGB1/Beclin1 complex formation. Nuclear(Nuc) and cytoplasmic(Cyt) extraction were performed in HepG2.2.15 and HBx‐L02 cells. Assay for HMGB1/Beclin1 interaction was indicated by co‐IP and western blotting. (B) Colocalization between Beclin1 and HMGB1 in liver samples of HBV‐infected patients was analyzed by confocal microscopy.

**Fig. S8.** (A) Schematic figures showing our knock‐in strategy for HBx allele using CRISPR/Cas9 by homologous recombination. The HBx‐transgenic mouse was verified by western blotting. (B) Immunoblot detecting indicated proteins in HBx‐transgenic mice and age‐matched WT mice.
