# Supplementary figures and images for: Crosstalk between hepatitis B virus X and high‐mobility group box 1 facilitates autophagy in hepatocytes
Source: Mol Oncol. 2018 Jan 24;12(3):322–38. doi: 10.1002/1878-0261.12165 (PMC5830655; doi:10.1002/1878-0261.12165)

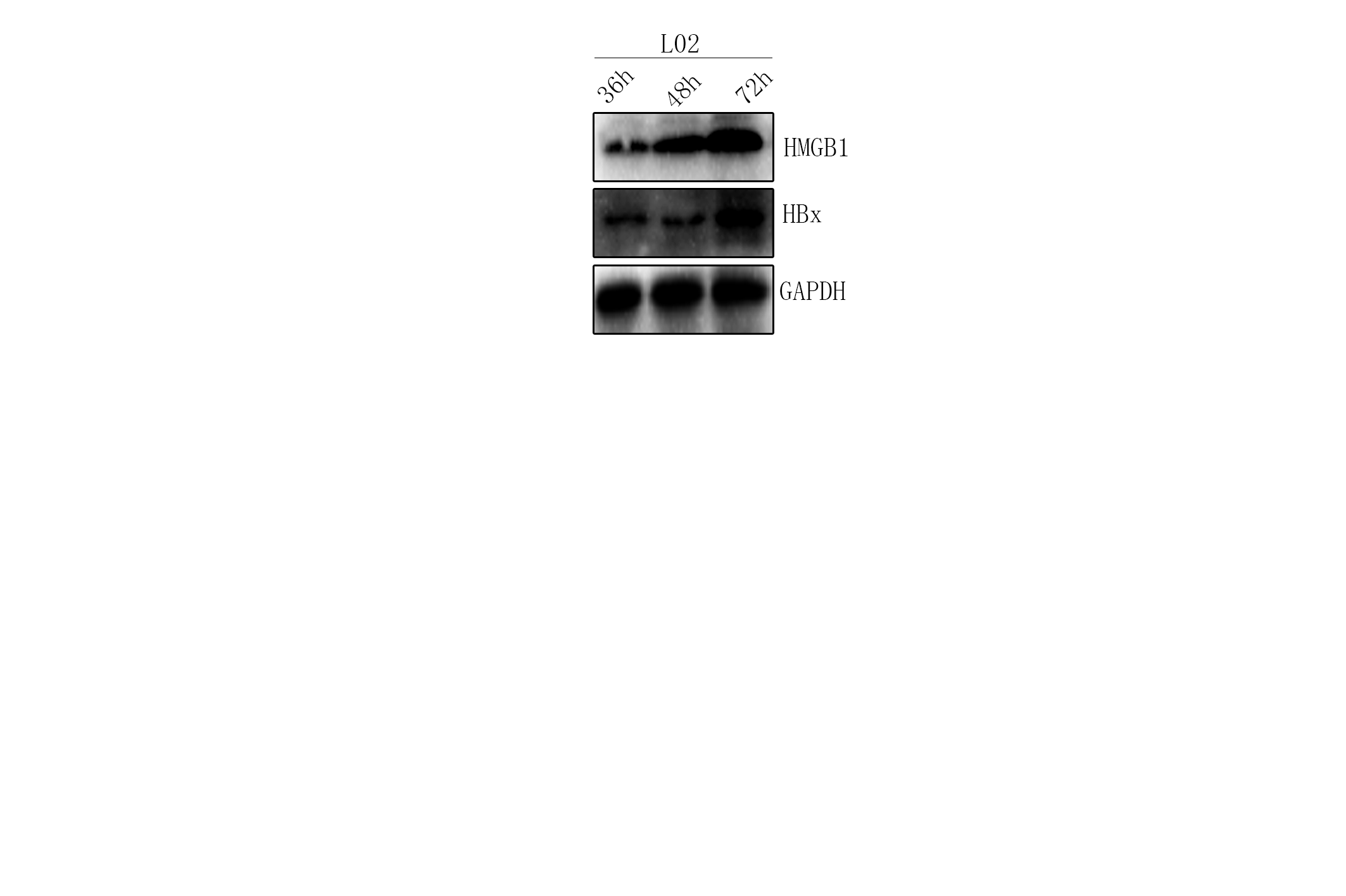

Supplement: Supplementary file 1 — Fig. S1. HMGB1 expression at the indicated time points after transfection with HBx(3 μg)in L02 cells was analyzed by Western blot. [file MOL2-12-322-s001.tiff]

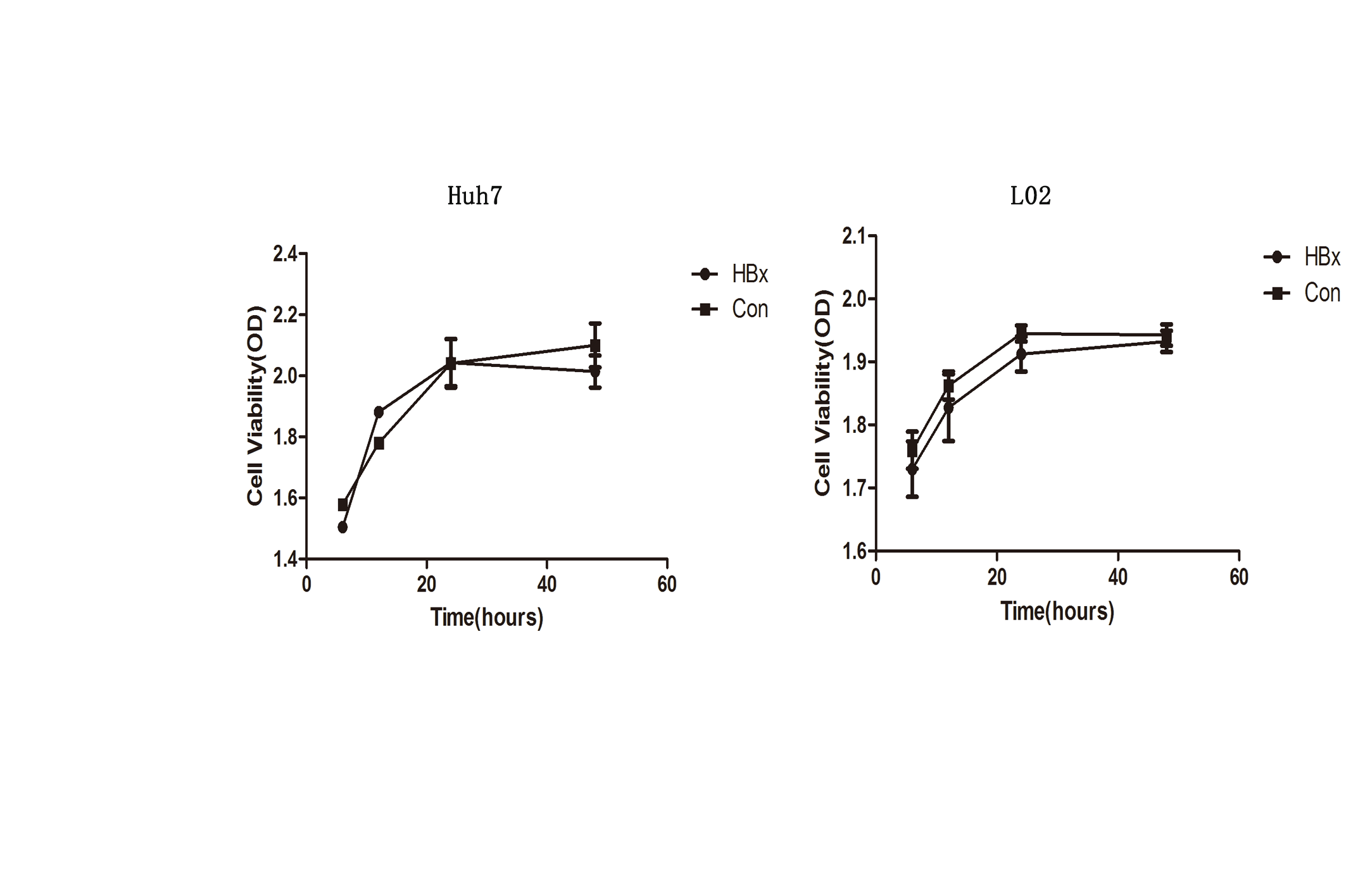

Supplement: Supplementary file 2 — Fig. S2. Cell viability was determined by CCK8 assay after cells (Huh7 and L02 )transfected with HBx(3 μg)at indicated time periods(48 h). [file MOL2-12-322-s002.tiff]

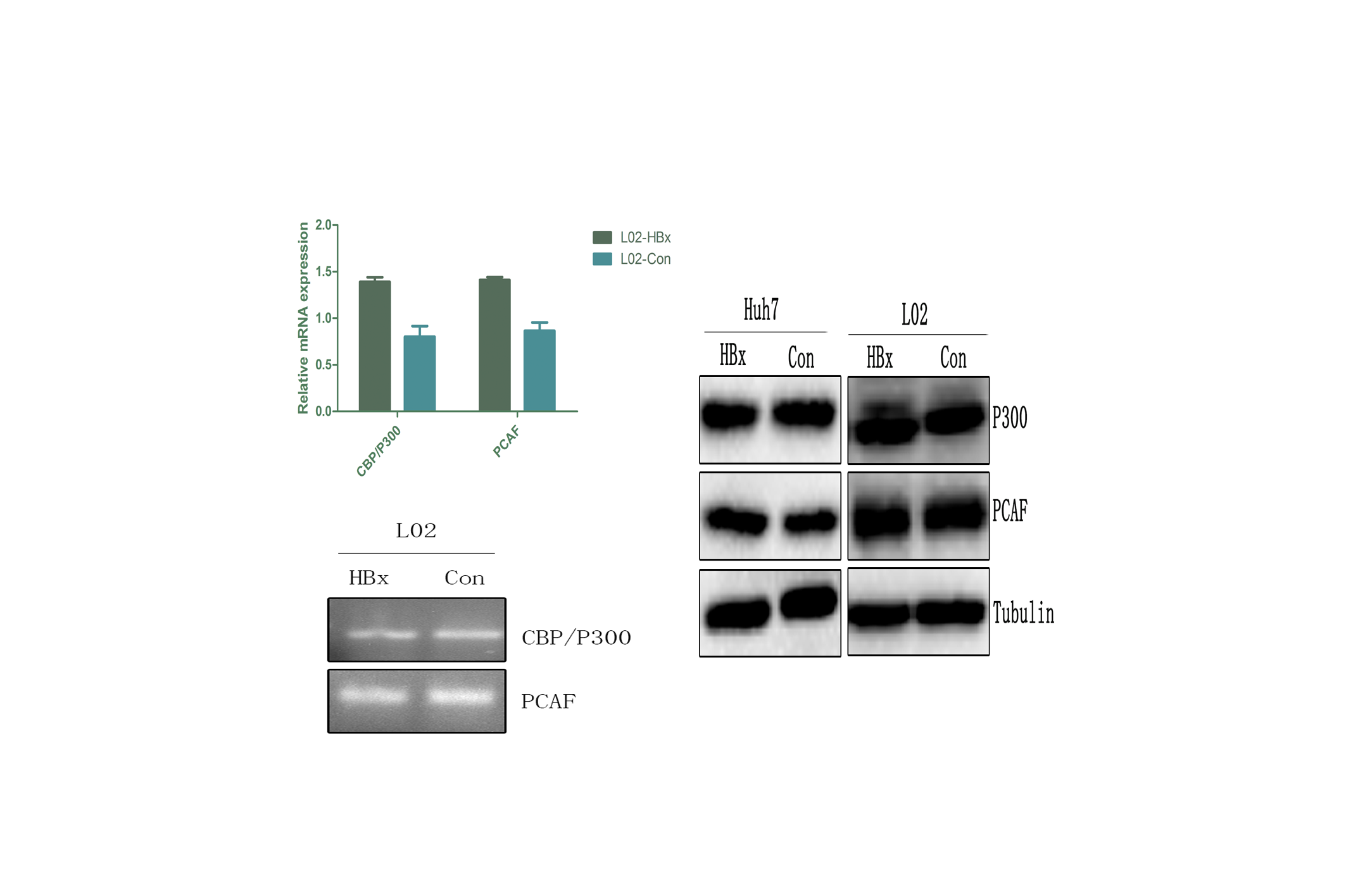

Supplement: Supplementary file 3 — Fig. S3. Expression levels of CBP/P300 and PCAF in HBx‐L02 and Vector‐L02 cells were detected by RT‐PCR and Western blot. [file MOL2-12-322-s003.tiff]

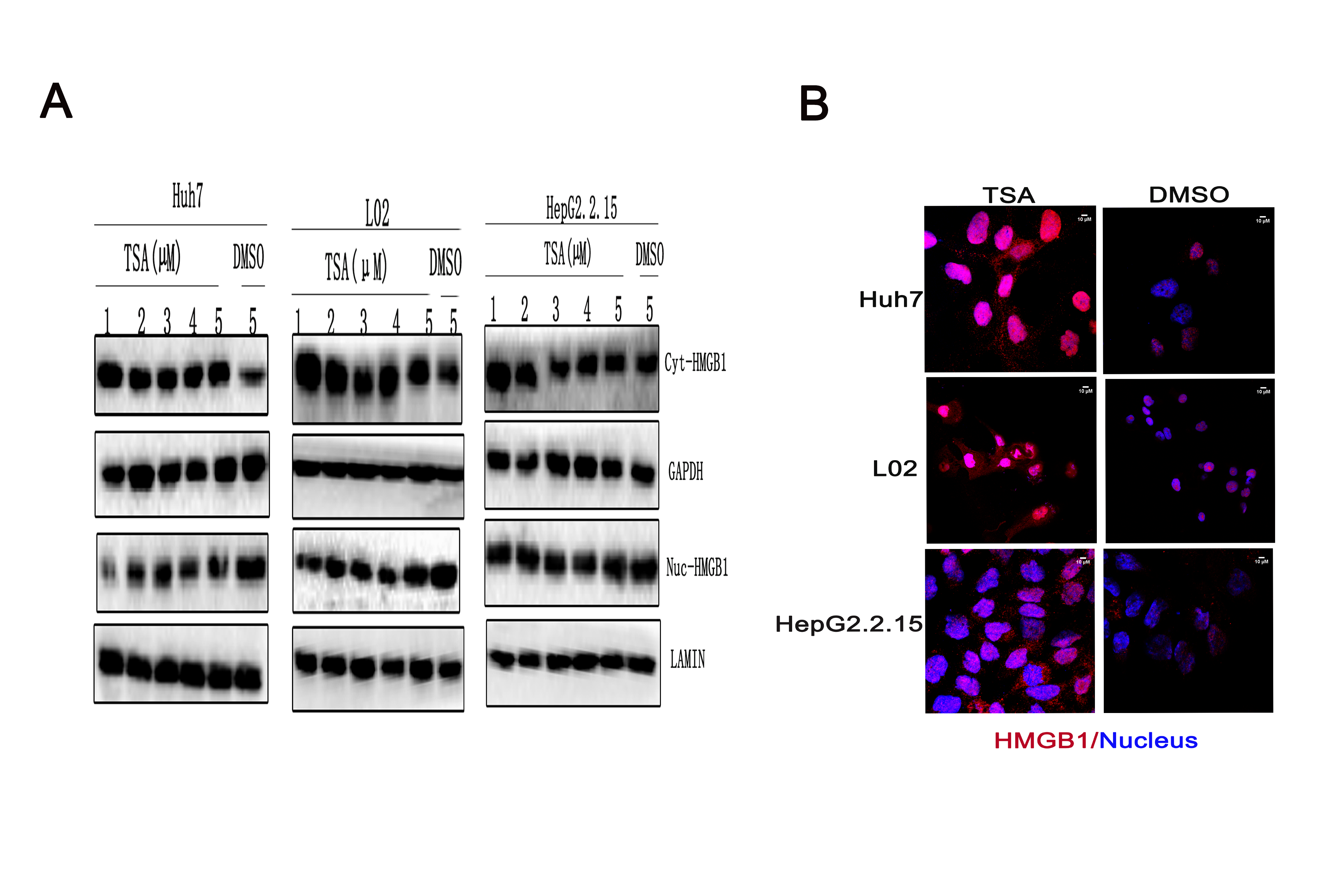

Supplement: Supplementary file 4 — Fig. S4. HDAC inhibition by TSA promotes the Cyt translocation of HMGB1. [file MOL2-12-322-s004.tiff]

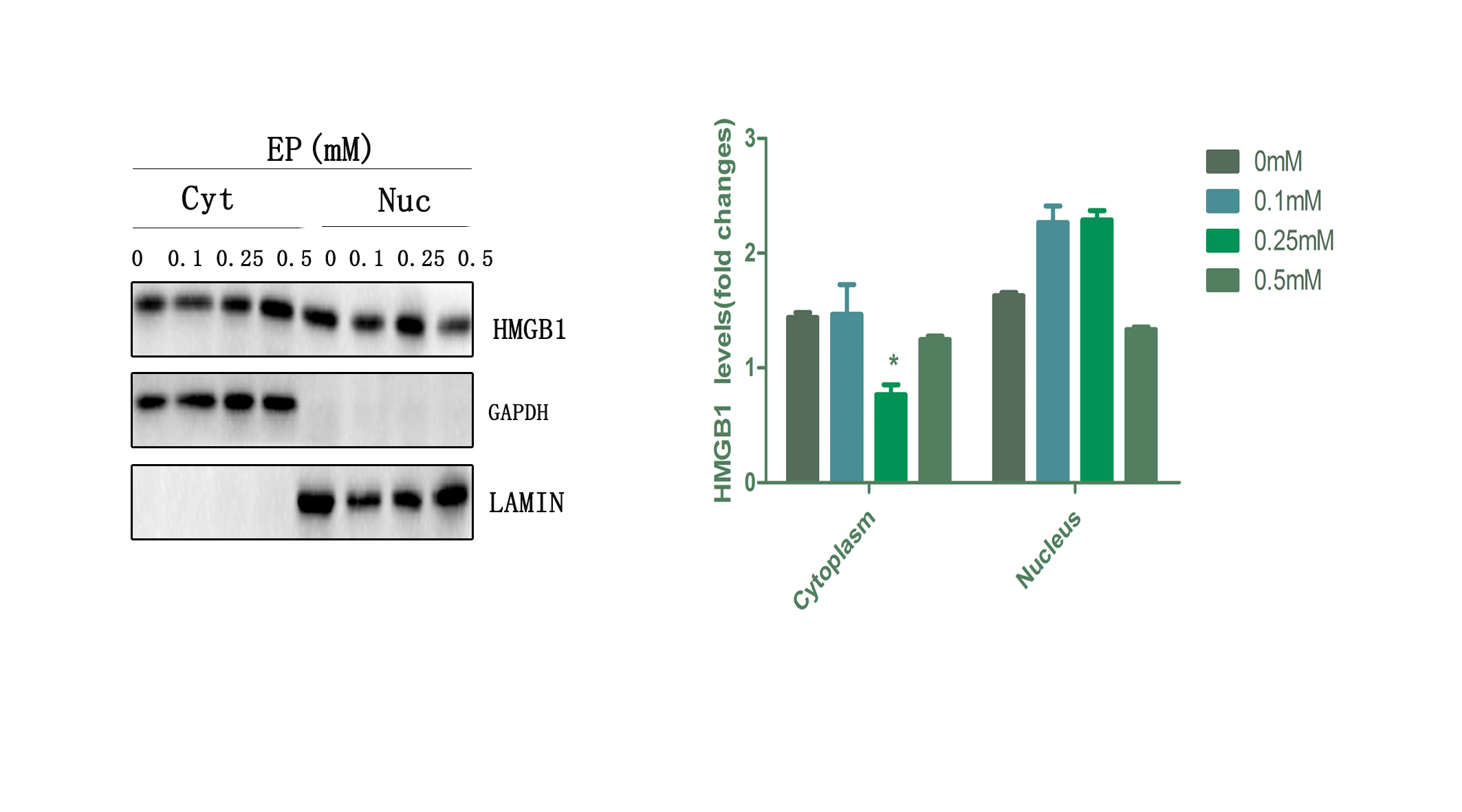

Supplement: Supplementary file 6 — Fig. S6. HBx‐L02 cells were treated with EP (0.1–0.5 mm) for 24 h. [file MOL2-12-322-s006.tiff]

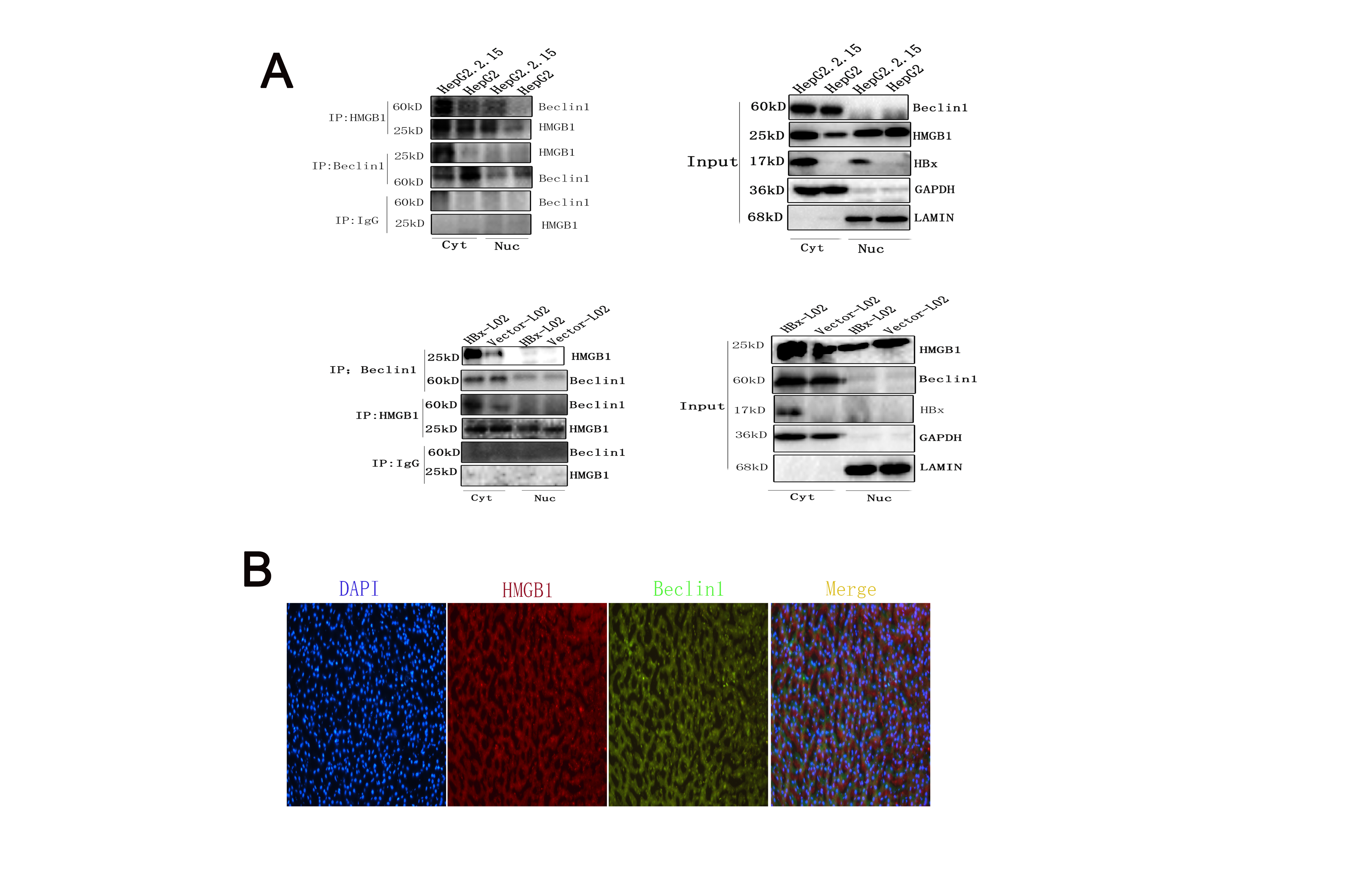

Supplement: Supplementary file 7 — Fig. S7. (A) HBx overexpression promotes HMGB1/Beclin1 complex formation. Nuclear(Nuc) and cytoplasmic(Cyt) extraction were performed in HepG2.2.15 and HBx‐L02 cells. Assay for HMGB1/Beclin1 interaction was indicated by co‐IP and western blotting. (B) Colocalization between Beclin1 and HMGB1 in liver samples of HBV‐infected patients was analyzed by confocal microscopy. [file MOL2-12-322-s007.tiff]

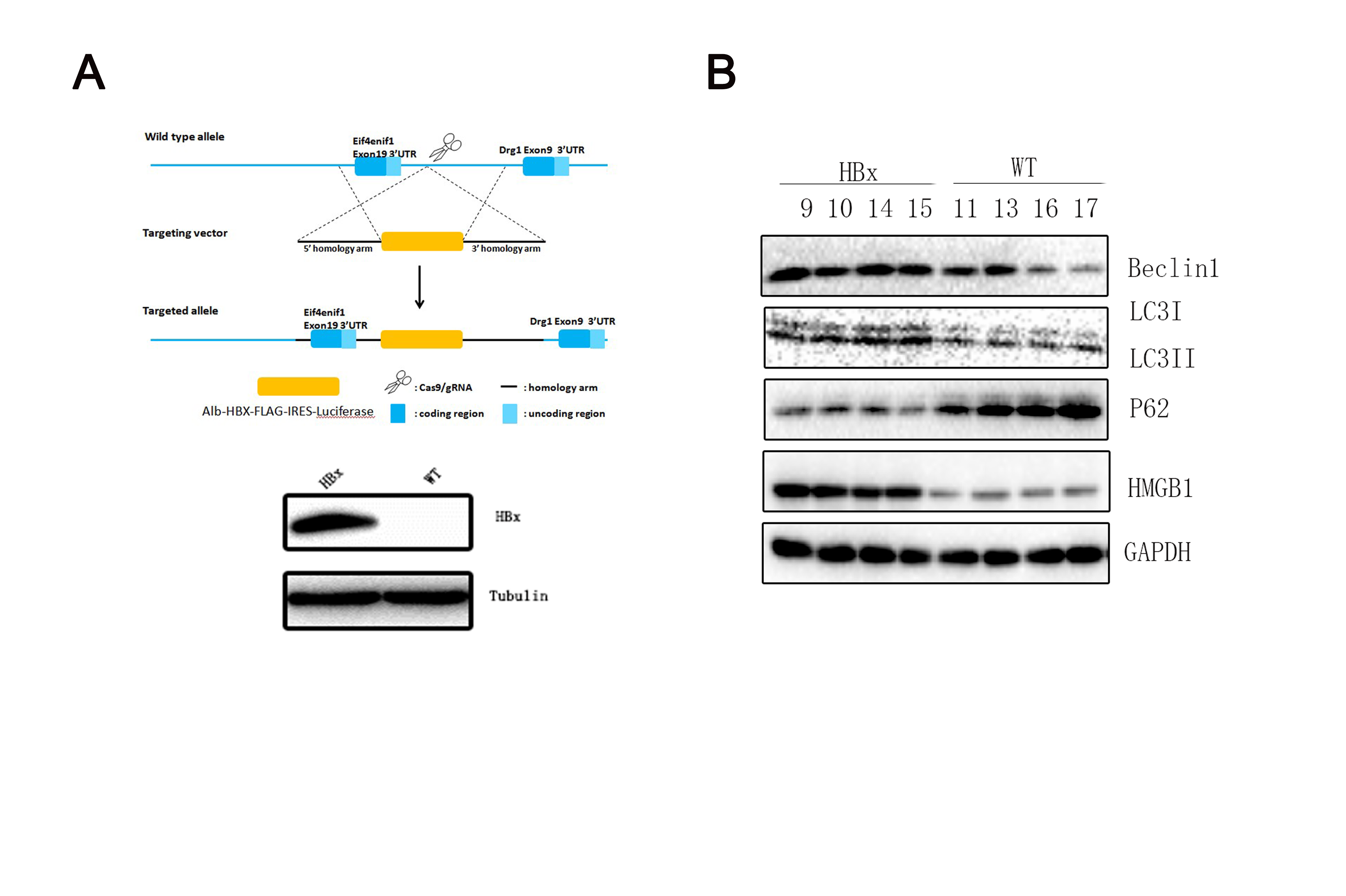

Supplement: Supplementary file 8 — Fig. S8. (A) Schematic figures showing our knock‐in strategy for HBx allele using CRISPR/Cas9 by homologous recombination. The HBx‐transgenic mouse was verified by western blotting. (B) Immunoblot detecting indicated proteins in HBx‐transgenic mice and age‐matched WT mice. [file MOL2-12-322-s008.tiff]
